# The non-photochemical quenching protein LHCSR3 prevents oxygen-dependent photoinhibition in *Chlamydomonas reinhardtii*

**DOI:** 10.1093/jxb/eraa022

**Published:** 2020-01-16

**Authors:** Thomas Roach, Chae Sun Na, Wolfgang Stöggl, Anja Krieger-Liszkay

**Affiliations:** 1 Department of Botany and Centre for Molecular Biology Innsbruck, Leopold-Franzens-Universität-Innsbruck, Sternwartestraße 15, Innsbruck, Austria; 2 Seed Conservation Research Division, Department of Seed Vault, Baekdudaegan National Arboretum, Munsu-ro, Chunyang-myeon, Bonghwa-gun, Gyeongsangbuk-do, Republic of Korea; 3 Institute for Integrative Biology of the Cell (I2BC), Commissariat à l’Energie Atomique et aux Energies Alternatives (CEA), Centre National de la Recherche Scientifique (CNRS), Université Paris-Sud, Université Paris-Saclay, Gif-sur-Yvette, France; 4 Bielefeld University, Germany

**Keywords:** Carboniferous, electrophile, evolution, non-photochemical quenching, photoinhibition, photosynthesis, reactive oxygen species, stress, qE

## Abstract

Non-photochemical quenching (NPQ) helps dissipate surplus light energy, preventing formation of reactive oxygen species (ROS). In *Chlamydomonas reinhardtii*, the thylakoid membrane protein LHCSR3 is involved in pH-dependent (qE-type) NPQ, lacking in the *npq4* mutant. Preventing PSII repair revealed that *npq4* lost PSII activity faster than the wild type (WT) in elevated O_2_, while no difference between strains was observed in O_2_-depleted conditions. Low *F*_v_/*F*_m_ values remained 1.5 h after moving cells out of high light, and this qH-type quenching was independent of LHCSR3 and not accompanied by losses of maximum PSII activity. Culturing cells in historic O_2_ atmospheres (30–35%) increased the qE of cells, due to increased LHCSR1 and PsbS levels, and LHCSR3 in the WT, showing that atmospheric O_2_ tensions regulate qE capacity. Colony growth of *npq4* was severely restricted at elevated O_2_, and *npq4* accumulated more reactive electrophile species (RES) than the WT, which could damage PSI. Levels of PsaA (PSI) were lower in *npq4* grown at 35% O_2_, while PsbA (PSII) levels remained stable. We conclude that even at high O_2_ concentrations, the PSII repair cycle is sufficient to maintain net levels of PSII. However, LHCSR3 has an important function in protecting PSI against O_2_-mediated damage, such as via RES.

## Teaser

At high O_2_ tensions, as encountered prehistorically, *Chlamydomonas reinhardtii* elevated levels of NPQ-related proteins and LHCSR3 had an important function in protecting PSI.

## Introduction

Molecular oxygen is a photosynthetic by-product from the water-splitting activity of PSII. Oxygen started accumulating in the atmosphere 2.4 billion years ago (bya) due to photosynthesis (Lyons, 2014), which enabled the evolution of oxidative phosphorylation, contributing to the evolution of sex and multicellular life (Hörandl and Speijer, 2018). Today, sunlight drives almost all primary productivity on this planet, and PSII is considered as ‘the engine of life’ (Barber, 2003). However, O_2_ also forms unstable radical and non-radical reactive oxygen species (ROS), and the initial accumulation of O_2_ is thought to have caused the first major extinction event of our planet (Lane, 2002). For plants, photosynthesis is a major source of ROS that have to be dealt with (Halliwell, 2006), especially in response to increasing light intensity (Roach *et al.*, 2015a).

Singlet oxygen (^1^O_2_) is a highly destructive ROS produced by PSII (Krieger-Liszkay, 2005; Fischer *et al.*, 2013; Telfer, 2014). In higher plants, very high levels of ^1^O_2_ activate programmed cell death (Op den Camp *et al.*, 2003). At lower and more typical physiological levels, ^1^O_2_ peroxidizes membrane lipids, which break down and release aldehydes that include reactive electrophile species (RES), such as acrolein (Fischer *et al.*, 2012; Mano, 2012; Roach *et al.*, 2017; Yalcinkaya *et al.*, 2019). Several studies have shown that ROS and RES contribute directly to PSII damage (Hideg *et al.*, 1994; Chan *et al.*, 2012; Roach *et al.*, 2013; Kale *et al.*, 2017), while other studies have shown that ROS inhibit the repair of the PSII reaction centres (Nishiyama *et al.*, 2001; Murata *et al.*, 2007). PSI is much more stable than PSII. Photoinhibition of PSI has only been reported for certain chilling-sensitive species and under fluctuating light for mutants affected in cyclic electron transport (e.g. *pgr5*, *pgrl1*, and *crr*). Mechanisms of PSI photoinhibition are not fully resolved, but ROS production has been shown to be involved (Takagi *et al.*, 2016). Importantly, photoinhibition of PSI is a very costly process for the plant since no fast repair cycle exists (Sonoike, 2011). The 4Fe4S clusters F_A_, F_B_, and F_x_ have been identified as the site of damage in PSI (Sonoike *et al.*, 1995; Tiwari *et al.*, 2016).

To protect against photoinhibition, photosynthetic organisms require ways of safely dissipating excess light energy. In part, this is achieved by non-photochemical quenching (NPQ) that regulates light energy use efficiency (Müller *et al.*, 2001). Mutants deficient in NPQ of the higher plant *Arabidopsis thaliana* and the chlorophytic green alga *Chlamydomonas reinhardtii* produce more ROS than comparative wild types (WTs) under high light (Havaux *et al.*, 2000; Baroli *et al.*, 2004; Roach and Krieger-Liszkay, 2012; Allorent *et al.*, 2013; Roach *et al.*, 2015a). Further to just dissipating excess energy and preventing ROS production, NPQ regulates photosynthetic electron flow, contributing to plant and algal growth (Cardol *et al.*, 2009; Tikkanen *et al.*, 2011; Kromdijk *et al.*, 2016).

NPQ consists of several processes, the pH-dependent component (qE), state transitions (qT), photoinhibition (qI), and a further type of sustained quenching (qH) that is not associated with qI. qE is rapidly inducible within seconds and is activated in response to a low pH in the thylakoid lumen (Horton *et al.*, 2005; Holzwarth *et al.*, 2009). In higher plants, the four-helix light-harvesting complex (LHC)-related PsbS protein is involved in qE (Li *et al.*, 2000). In *C. reinhardtii* and the moss *Physcomitrella patens*, the PsbS protein also contributes to qE (Alboresi *et al.*, 2010; Correa-Galvis *et al.*, 2016; Tibiletti *et al.*, 2016), alongside three-helix LHC-type proteins called ‘light-harvesting-complex-stress-related’ (LHCSR) that are absent in higher plants. Under increased light exposure, LHCSR expression levels are up-regulated, which is in contrast to the typical down-regulation of other LHC genes (Büchel, 2015; Niyogi and Truong, 2013). *Chlamydomonas reinhardtii* has two closely related genes, *LHCSR3.1* and *LHCSR3.2*, that both encode the same LHCSR3 protein, and LHCSR1 that is 82% identical to LHCSR3 (Peers *et al.*, 2009). *LHCSR3.1*, *LHCSR3.2*, and *LHCSR1* genes have minor differences in their sequence and promoter regions, leading to distinct regulation (Maruyama *et al.*, 2014). While blue light is stronger in up-regulating *LHCSR3.1* and *LHCSR3.2* (Petroutsos *et al.*, 2016), UV-B radiation strongly up-regulates *LHCSR1* and *PSBS*, whose corresponding proteins provide photoprotection (Allorent *et al.*, 2016; Dinc *et al.*, 2016; Tilbrook *et al.*, 2016). Of the various *C. reinhardtii* NPQ mutants, including *npq1* (deficient in violaxanthin deepoxidase and therefore in antheraxanthin and zeaxanthin), *npq2* (deficient in zeaxanthin epoxidase), *stt7-9* (deficient in STT7 kinase and therefore state transitions), and *lhcsr1* (deficient in LHCSR1), the *npq4* mutant (deficient in LHCSR3) has the lowest qE capacity (Niyogi *et al.*, 1997; Peers *et al.*, 2009; Allorent et al., 2013, 2016). Dissipating excess light energy via LHCSR3 involves protonation of luminal residues at the C-terminus, connecting the low pH of the thylakoid lumen to activation of qE (Bonente *et al.*, 2011; Liguori *et al.*, 2013; Ballottari *et al.*, 2016). However, LHCSR deficiency does not necessarily lead to PSII photoinhibition under constant light approaching a saturating intensity (Cantrell and Peers, 2017). LHCSR3 has been identified in the PSI antenna of *C. reinhardtii* (Allorent *et al.*, 2013; Bergner *et al.*, 2015), where it may potentially quench the excitation energy of LHCII, thereby decreasing the excitation pressure of PSI, as shown in the moss *P. patens* (Pinnola *et al.*, 2015) and *C. reinhardtii* (Girolomoni *et al.*, 2019). Photoinhibition of PSI occurred within a few hours of high light treatment in the *C. reinhardtii pgrl1npq4* double mutant, deficient in LHCSR3 and *pgrl1*-mediated cyclic electron flow, but not in the *pgrl1* single mutant (Bergner *et al.*, 2015; Chaux *et al.*, 2017). Overall, this indicates that LHCSR3 can protect PSI from photodamage.

Historically, atmospheric oxygen peaked ~0.3 bya in the carboniferous period at a level of 30–35% (Holland, 2006), and algae of this period may have benefited from a complexity of photoprotective qE mechanisms (LHCSR1, LHCSR3, and PsbS). Despite current atmospheric levels of 21%, the water column that algae inhabit can become highly oxygenated at peak light intensities due to high photosynthetic rates (Roach *et al.*, 2015b).

The aim of this study was to investigate the importance of LHCSR3 for *C. reinhardtii* to cope with the combination of elevated O_2_ and high light. We used the LHCSR3-deficient *npq4* mutant of *C. reinhardtii* alongside two WT strains: WT-4A, the WT parent of *npq4*; and WT-D66 that has higher LHCSR3 levels than WT-4A. Cells were cultivated photoautotrophically in O_2_ tensions putatively encountered ~0.3 bya (35% O_2_) and compared with cells at a lower O_2_ tension (17% O_2_). LHCSR3 could protect both PSII and PSI from O_2_-dependent damage but, due to efficient repair of PSII, only PSI levels decreased in *npq4* in 35% O_2_. Tolerance to ^1^O_2_, and levels of LHCSR1 and RES were elevated in *npq4*, particularly in cells cultivated in 35% O_2_. Since the RES acrolein strongly up-regulated *LHSCR1*, alongside transcription of many other light stress-associated genes, we discuss the Jekyll and Hyde nature of RES, which on the one hand contribute to retrograde signalling, leading to elevated qE capacity, while on the other hand cause damage, including photoinhibition.

## Materials and methods

### Strains and growth conditions

*Chlamydomonas reinhardtii* WT-4A^+^ (CC-4051) *npq4*^*+*^ (CC-4614; positive mating type *npq4*) were used in all experiments, and, when indicated, *npq4*^−^ (CC-4615; negative mating type *npq4*) was also included. Strains were purchased from the Chlamydomonas Centre (www.chlamycollection.org). When indicated, the WT strain D66 (CC-4425) was also used (a gift from L. Michelet, CEA Saclay, France). Cultures were initiated in Tris-acetate-phosphate (TAP) liquid medium, pH 7.0, and grown photoheterotrophically under low light (50 µmol photons m^−2^ s^−1^). To transfer cells to photoautotrophic conditions, TAP cultures were pelleted for 2 min at 1600 *g* and resuspended in Tris-HCl-phosphate (THP) medium (identical except the pH was adjusted to 7.0 with HCl rather than acetic acid) and cultivated under low light while being bubbled with sterile air, using a 0.22 µm air filter. Cells were in THP for at least 24 h before experiments began, which is well beyond the time for residual acetate to be consumed that can affect ^1^O_2_ production by PSII (Roach *et al.*, 2013). Liquid cultures were rotated at 80 rpm at 20 °C, kept in the exponential growth phase, and adjusted to 10 µg chlorophyll ml^−1^ before starting each experiment. Chlorophyll was measured according to Porra *et al.* (1989) in 80% acetone.

### Elevated oxygen growth tests

A 10 µl aliquot of TAP cultures at 1×10^6^ cells ml^−1^ was spotted onto THP medium containing 1.5% agar and the medium was dried off in a sterile air flow over 0.5 h. The agar was transferred onto a plastic insert that was held in the neck of an upside down 1 litre clear glass jar. The O_2_ content of the jar was increased with pure O_2_ gas to the desired concentration, as measured with O_2_ optode sensor spots (PreSens, Regensburg, Germany) placed on the inside of the sealed jars. The sensors were calibrated with pure O_2_ and N_2_ gases. Jars were placed in an incubator at 25 °C and 250 µmol photons m^−2^ s^−1^ on a 16/8 h (day/night) diurnal cycle for 7 d. The lids were opened after 3 d and gases exchanged. In a subsequent experiment for LHCSR1, LHCSR3, and PsbS protein analyses, cells were cultivated as above, except that the O_2_ level was adjusted to 35% and 17% using pure O_2_ and N_2_, respectively, so that gas displacement led to the same CO_2_ levels (0.033%) in both conditions. Cells were removed for analyses 6–8 h after the onset of light.

### High light and gas treatments of liquid cultures

High light was provided by a 250 W horticultural compact fluorescent lamp (Envirolite, 6400K) and cultures were kept between 20 °C and 25 °C with fan-assisted cooling. The light intensity measured at the top and bottom of liquid cultures was 300 µmol photon m^−2^ s^−1^ and 200 µmol photon m^−2^ s^−1^, respectively (from here on 250 µmol photon m^−2^ s^−1^), which was a 5-fold increase over the growth light intensity. Liquid cultures were pre-high light treated for 2 h in the absence of air bubbling to induce the production of LHCSR3 in WT cells, and then recovered for 2 h at 30 µmol photons m^−2^ s^−1^ to enable recovery of any photoinhibitory effects of the pre-high light treatment. After this, the *F*_v_/*F*_m_ of WT-4A, WT-D66, and *npq4* were 0.63±0.01, 0.65±0.02, and 0.61±0.01, respectively, and net O_2_ production rates under saturating light (PSII activity) were 218±31, 169±8, and 149±32 µmol mg^−1^ chlorophyll h^−1^, respectively (*n*=3±SD). Subsequently, for measuring rates of photoinhibition, cells were re-treated with high light in the presence of 2.5 mM lincomycin and in the presence of 5 mM NaHCO_3_, either constantly bubbled with either N_2_ or O_2_ gas, or without any gas bubbling, as indicated in the figure legends.

### Photosynthetic measurements

Net O_2_ production at saturating light intensity (1500 µmol photons m^−2^ s^−1^) was measured using a Fibox 3 optode dipping probe (PreSens, Regensburg, Germany) in the presence of 1 mM NaHCO_3_ with constant stirring. For measuring chlorophyll fluorescence of liquid cultures, a cuvette-adapted Aquapen-C was used (Photon System Instruments, Drasov, Czech Republic). A 2 ml aliquot of culture was diluted to 0.5 µg ml^−1^ chlorophyll, and *F*_o_ (background fluorescence) and *F*_m_ (maximum fluorescence) were measured before and during a 2 s saturating pulse of 3000 µmol photons m^−2^ s^−1^. Maximum and relative quantum yields of PSII (*F*_v_/*F*_m_ and ФPSII, respectively) were calculated via (*F*_o_–*F*_m_)/*F*_m_, whereby *F*_m_ was measured before (ФPSII) or after (*F*_v_/*F*_m_) 1.5 h dark recovery to allow the majority of *F*_m_ quenching to relax, while maintaining cells in the physiological state closest to that at the end of the light treatment. qE was measured using the NPQ_1 program (2 min at 1000 µmol photons m^−2^ s^−1^) and calculated with (*F*_o_–*F*_m_')/*F*_m_', with *F*_m_' measured during actinic light. For measuring chlorophyll fluorescence of agar-grown cultures, a CCD camera (FluorCam 701MF, Photon System Instruments) was used. *F*_m_ was measured with a 600 ms saturating pulse of 2300 µmol photons m^−2^ s^−1^. For measuring qE, actinic light was provided by two red LED panels, providing 200 µmol photons m^−2^ s^−1^ and measured after 2 min light treatment. The redox state of PSI was measured by near infra-red absorption with a Dual-PAM-100 (Heinz Walz, Effeltrich, Germany) according to the method of Klughammer and Schreiber (1994). The maximum P700 change from the dark-adapted to fully oxidized level (P700^+^) obtained 20 ms after a saturating light pulse was calculated manually in Excel after exporting raw data as the difference between 0–5 ms before and 19–21 ms after starting the saturating pulse. Cultures with a chlorophyll content of 80 µg ml^−1^ were dark adapted for 2 h and vigorously stirred immediately before measurement. A minimum of three technical replicate measurements were averaged for each biological replicate.

### Analysis of LHCSR3, LHCSR1, PsbS, PsaA, and PsbA protein levels

Proteins were extracted from the same cells, or pool of cells, and non-invasively measured (e.g. chlorophyll fluorescence) for data that are shown within the same figure, or related supplementary figure. Total cellular proteins were either extracted in 2% SDS, in 50 mM Tris-HCl, pH 6.8 (for agar-grown cultures), or in 7 M urea, 2 M thiourea, 20 mM Tris–HCl with 0.2% (v/v) Triton X-100 (for liquid cultures), in both cases with protease inhibitor cocktail (Complete Mini, Roche Diagnostics, Switzerland), with protein quantification via the bicinchoninic acid or Bradford assay, for the two extraction methods, respectively. Before loading, proteins were denaturated at 85 °C with 0.1 M DTT and separated by PAGE using 12% acrylamide gels at 40 mA in Tris-glycine-SDS running buffer. For semi-dry western blotting, separated proteins were transferred to nitrocellulose membranes at 40 mA per gel for 1 h, which were subsequently blocked in 5% fat-free milk powder before incubating with either LHCSR3 (AS14-2766), LHCSR1 (AS14-2819), or PsaA (AS06-172) antibodies at 1:10 000 dilution, PsbA antibody (AS05-084) at 1:50 000 dilution (Agrisera, Sweden), or PsbS (a gift from Stefano Caffarri, Aix Marseille University) at 1:2000 dilution, for 1 h at room temperature. The peroxidase-coupled secondary antibodies (Sigma-Aldrich, St Louis, MO, USA) were visualized with enhanced chemiluminescence (Amersham, GE Healthcare, UK) and light-sensitive film (Amersham, GE Healthcare, UK). Blots were scanned for densitometry in ImageJ (Schneider *et al.*, 2012).

### Quantification of RES and other aldehydes via LC-MS/MS

Aldehydes were measured according to Roach *et al.* (2018). Briefly, cultures grown on agar were carefully scraped off the agar, weighed, and immediately suspended in 1 ml of acetonitrile with 0.5 µM 2-ethylhexanal (as internal standard) and 0.05% (w/v) of butylated hydroxytoluene. After centrifugation, aldehydes in the supernatant were derivatized with 2,4-dinitrophenylhydrazine (DNPH) in the presence of formic acid and diluted 50:50 with ultra-pure H_2_O before injection. Separation was carried out using a reversed-phase column (NUCLEODUR C18 Pyramid, EC 50/2, 50×2 mm, 1.8 µm, Macherey-Nagel, Düren, Germany), with an ekspert ultraLC 100 UHPLC system (AB SCIEX, Framingham, MA, USA) coupled to a QTRAP 4500 mass spectrometer for quantification of 2,4-DNPH-RES. Peak areas of selected ions were normalized relative to the internal standard, and concentrations were calculated to absolute amounts according to the calibration curves using external standards, which were treated and derivatized in the same way as samples. For other aldehydes that were not injected as external standards, peak areas of the DNPH-aldehyde were normalized to dry weight and shown as relative levels, as shown for the WT and *npq4* cultured in 17% and 35% O_2_.

### Singlet oxygen resistance test

Resistance to ^1^O_2_ was performed according to Fischer *et al.* (2012). Briefly, 1 ml of liquid culture at 2×10^6^ cells ml^−1^ was pipetted into a 24-well flat-bottomed culture plate and Rose Bengal was added to the final concentrations indicated. Chlorophyll contents were used as an indicator of cell growth after 24 h at 50 μmol photons m^−2^ s^−1^. Data were normalized to the amount of chlorophyll in the absence of Rose Bengal for each interval of high light treatment and for WT and *npq4* separately. In another experiment, a similar Rose Bengal treatment was given to cells pre-exposed to high light or high light in 80% O_2_. After 4 min intervals, 10 µl of culture was pipetted onto TAP agar and cultivated for 5 d at low light intensity <10 µmol m^−2^ s^−1^.

### Statistics

Statistical analysis of data was carried out with the SPSS software package (v. 23) via one- or two-way ANOVA using Tukey’s post-hoc test. Univariate ANOVA-derived *P*-values for differences between factors or treatments are either given in the figures or, for simplicity, denoted by different letters above bars or symbols when *P*<0.05.

## Results

### LHCSR3 enables growth in an oxygenated atmosphere

**Fig. 1.**
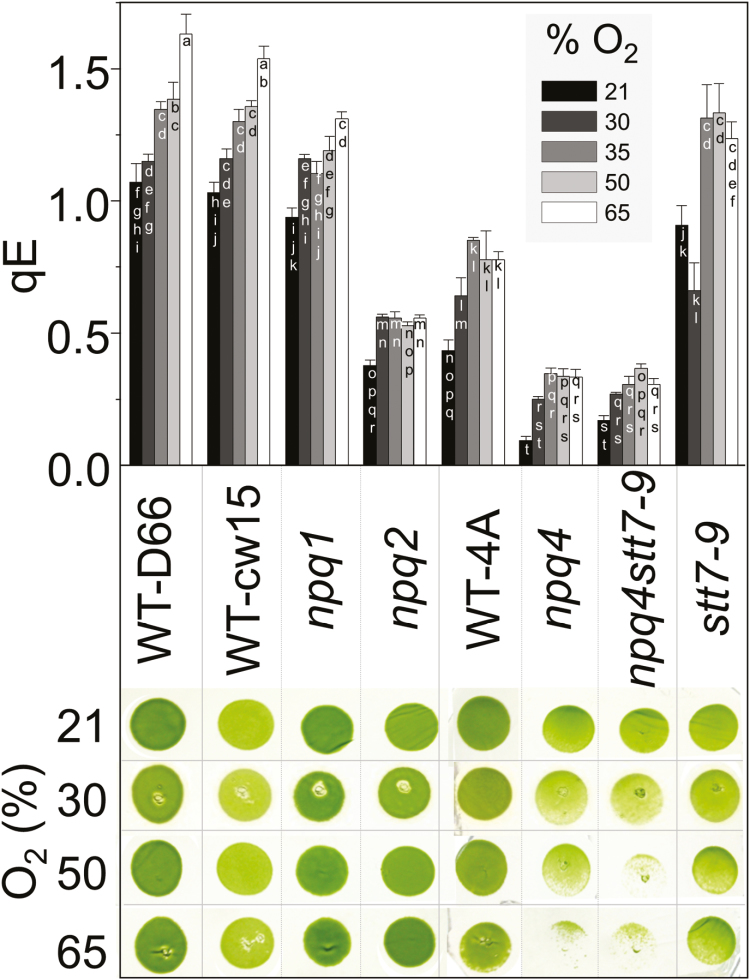
*C. reinhardtii* mutants deficient in LHCSR3 show sensitivity to culture initiation in elevated O_2_. Mutant strains (see text for details) and corresponding WTs were cultured for 3 d on 1.5% agar medium in photoautotrophic conditions in 21–65% O_2_ under continuous light (250 µmol photons m^−2^ s^−1^). Before measurement of qE, cells were allowed to recover in the dark for 1.5 h, *n*=3±SD. Colony growth was imaged after a subsequent 3 d culturing at 100 µmol photons m^−2^ s^−1^ in 21% O_2_. (This figure is available in colour at *JXB* online.)

**Fig. 2.**
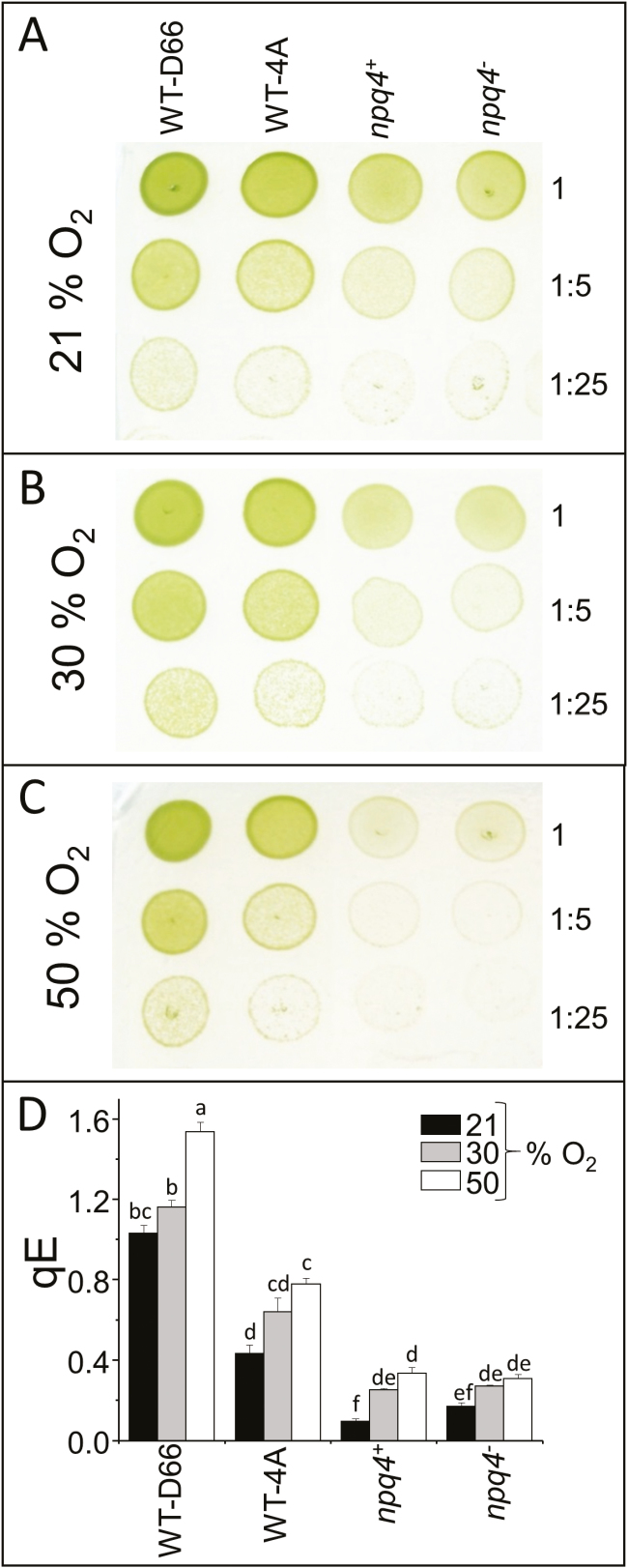
Higher qE capacity permits faster colony growth in elevated O_2_. WT-D66 (high LHCSR3), WT-4A (medium LHCSR3), and LHCSR3-deficient *npq4*^*+*^ and *npq4*^−^ were cultured on 1.5% agar medium in photoautotrophic conditions for 7 d under 250 µmol photons m^−2^ s^−1^, and a 16/8 h light/dark cycle, in (A) 21, (B) 30, or (C) 50% O_2_ environments. Before growth, culture spots, initiated from 10 µl of liquid culture at 15 µg ml^−1^ chlorophyll, were non-diluted or diluted 5-fold or 25-fold, as indicated to the right. (D) qE of cells after 7 d growth shown in (A–C). Before measurement of qE, cells were allowed to recover in the dark for 1.5 h, *n*=3±SD. (This figure is available in colour at *JXB* online.)

*Chlamydomonas reinhardtii* mutants affected in NPQ (*npq1*, *npq2*, *npq4*, *stt7-9*, and the *npq4stt7-9* double mutant) with their corresponding WTs, WT-cw15 (*npq1*, *npq2*, and *stt7-9*) and WT-4A (*npq4*), as well as WT-D66, were screened for the effect of elevated O_2_ on growth and qE. All strains increased qE capacity in elevated O_2_, but only LHCSR3-deficient mutants had growth-sensitive phenotypes (Fig. 1). Three of the strains were selected to further investigate the relationship between colony growth in elevated O_2_ and qE capacity: *npq4*, its parental WT (WT-4A), and WT-D66 that had the highest qE capacity (Fig. 1). After 7 d, colony growth of *npq4* was slowed in an atmosphere of 30% O_2,_ whereas growth of WT-4A and WT-D66 was hardly affected (Fig. 2A, B). In a constant atmosphere of 50% O_2_, colony growth of WT-D66 was equal to growth at 21% O_2_, whereas WT-4A was marginally less and *npq4* was considerably weakened (Fig. 2C). In agreement with Fig. 1, the qE capacity of all strains was increased by growth in elevated O_2_, but the differences between the genotypes were maintained (Fig. 2). At all O_2_ tensions, the qE of *npq4* was between 2- and 2.5-fold lower than of WT-4A (Figs 1, 2).

### Prevention of PSI photoinhibition in an oxygenated environment by LHCSR3

**Fig. 3.**
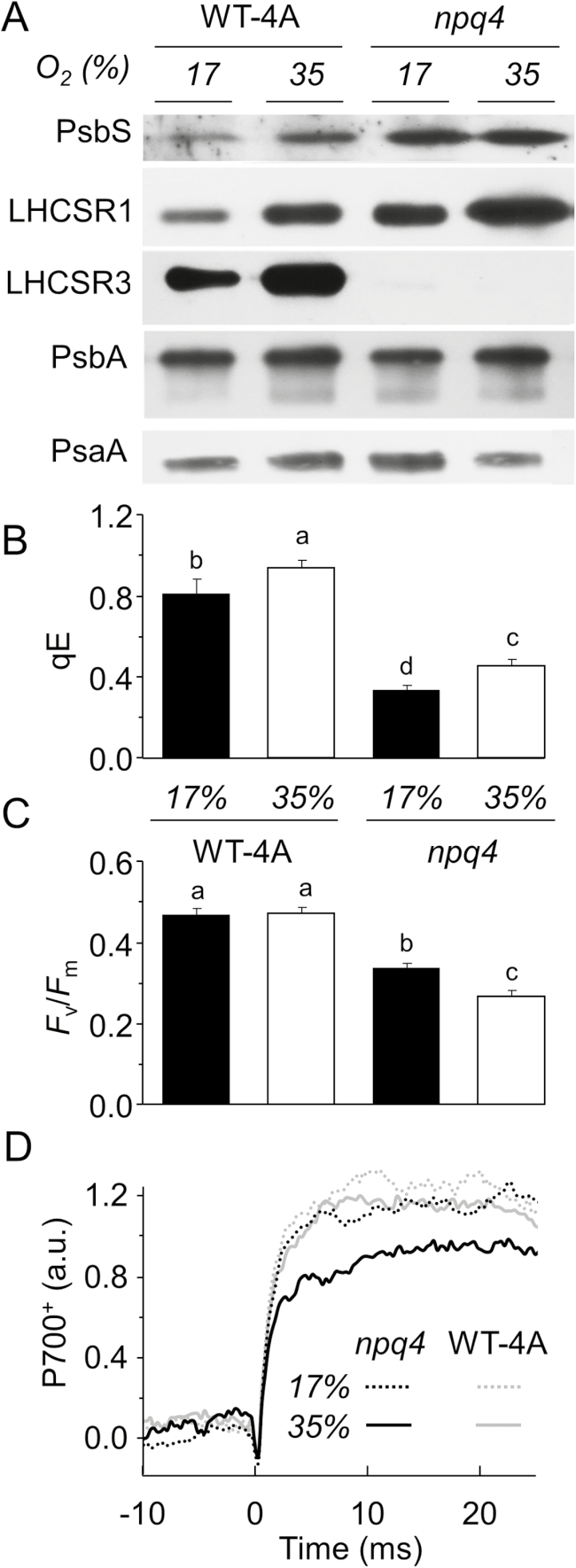
Growth in elevated O_2_ raises qE-related protein levels and qE capacity, but lowers PsaA and maximum P700^+^ levels in *npq4*. WT-4A and *npq4* were grown on 1.5% agar medium under photoautotrophic conditions in a 17% or 35% O_2_ atmosphere for 5 d under a 16/8 h light/dark cycle (250 µmol photons m^−2^ s^−1^). (A) The same protein extracts were used for all five blots. (B and C) Measurements of chlorophyll fluorescence were made after 1.5 h low light recovery, *n*=4±SD with different letters denoting significant differences (*P*<0.05). (D) P700-dependent absorption changes in WT-4A and *npq4* (see key). The increase in signal during a saturating pulse starting at time 0 corresponds to accumulation of P700^+^. Kinetics were averaged from four biological replicates each measured four times.

In order to address the potential relevance of the O_2_-dependent increase in qE capacity, WT-4A and *npq4* were cultivated on agar in 35% or 17% O_2_, in either case with equal CO_2_ availability of 0.033%. Cultivating WT-4A in 35% O_2_ increased levels of LHCSR1, LHCSR3, and PsbS compared with cultivation in 17% O_2_ (Fig. 3A). In *npq4*, PsbS and LHCSR1 were higher than in the WT at either 35% or 17% O_2_ (Fig. 3A), indicating compensation for the absence of LHCSR3. In *npq4*, the amount of LHCSR1 was higher in 35% O_2_ than in 17% O_2_. Noticeably, qE values of WT-4A and *npq4* were higher under 17% than 21% O_2_ (Figs 2, 3B), which is attributed to lower CO_2_ levels (0.033%) in 17% O_2_, due to gas displacement by adding N_2_ to lower O_2_ levels. Low CO_2_ elevates transcription of *LHCSR1* and *LHCSR3.1* (Maruyama *et al.*, 2014). Importantly, however, CO_2_ levels were equal in 17% and 35% O_2_ treatments. Densitometric quantifications of band intensity of western blots from three independent experiments showed no influence of O_2_ concentration on levels of the PSII reaction centre, as shown by the amount of PsbA in either genotype (Supplementary Fig. S1 at *JXB* online), despite lower *F*_v_/*F*_m_ values found in *npq4* (Fig. 3B). However, levels of the PSI reaction centre, as shown by the amount of PsaA, were on average, 26% lower in *npq4* cultivated in 35% O_2_, relative to cultivation in 17% O_2_, whereas PsaA levels in the WT were less altered by the O_2_ concentration (Supplementary Fig. S1). This agreed with only *npq4* grown in 35% O_2_ having lowered maximum P700^+^ levels (Fig. 3D). In summary, *C. reinhardtii* adjusted to oxygenated atmospheres by increasing its qE capacity via producing LHCSR1, LHCSR3, and PsbS. In contrast, *npq4*, despite increasing LHCSR1 and PsbS, showed a lower level of PsaA when grown in 35% O_2_.

### Prevention of PSII photoinhibition in an oxygenated environment by LHCSR3

**Fig. 4.**
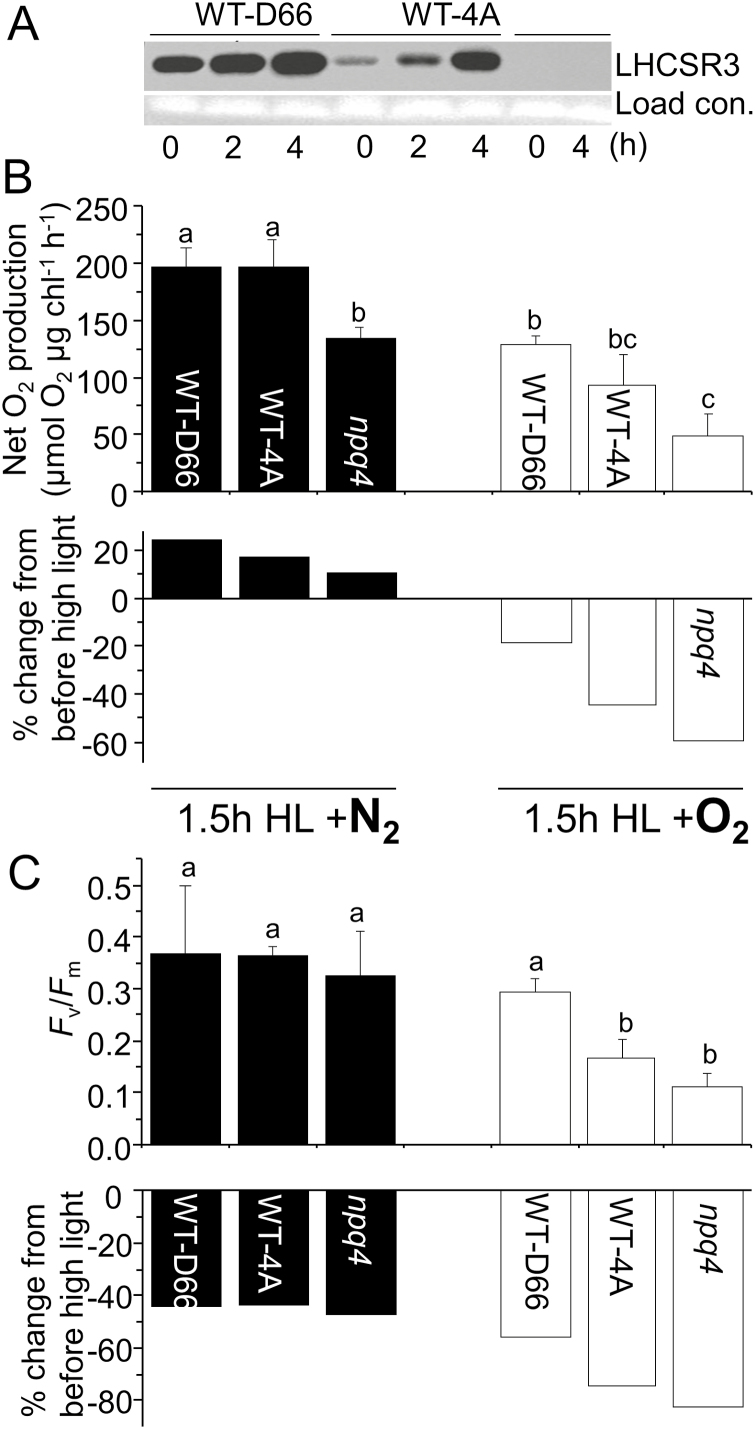
Oxygen accelerates PSII photoinhibition depending on LHCSR3 amounts. (A) Influence of high light (250 µmol photons m^−2^ s^−1^) on LHCSR3 levels in WT-D66, WT-4A, and *npq4.* High light-acclimated cultures were further high light treated for 1.5 h in the presence of 2.5 mM lincomycin and 5 mM NaHCO_3_ while purging with pure N_2_ (left panel; black) or pure O_2_ (right panel; white). PSII activity was measured after 1.5 h recovery by (B) O_2_ production at saturating light in the presence of 1 mM NaHCO3, and (C) *F*_v_/*F*_m_, *n*=3±SD. Data are from three independent experiments, with different letters denoting significant differences (*P*<0.05). The percentage changes from before to after high light treatment are indicated beneath (B) and (C).

For investigating the protection LHCSR3 affords against O_2_-mediated photoinhibition of PSII, liquid cultures were used, enabling the use of lincomycin to block PSII repair. Without lincomycin, high light (250 µmol photons m^−2^ s^−1^) treatment of WT and *npq4* photoautotrophic cultures in ambient O_2_ led to a steady increase in maximal PSII activity (from here on referred to as PSII activity), as monitored by net O_2_ production under saturating light without any CO_2_ restriction (Supplementary Fig. S2). In the presence of lincomycin, PSII activity was lost within 2 h high light (Supplementary Fig. S2), but there were limited differences between *npq4* and WT-4A. Therefore, in the next experiment, cultures were pre-treated with high light for 2 h to induce accumulation of LHCSR proteins. After this pre-treatment, LHCSR1 became detectable, and in a higher amount in *npq4* than in WT-4A (Supplementary Fig. S3), while WT cells accumulated more LHCSR3 (Fig. 4A). After allowing cultures to recover from the first high light treatment, cells were further treated with 1.5 h high light with lincomycin. In these conditions, losses of PSII activity in the three strains were prevented when the culture was constantly purged with N_2_ gas during high light treatment (Fig. 4B), in agreement with no loss of PsbA in the WT or *npq4* (Supplementary Fig. S4). In contrast, purging cultures with O_2_ gas significantly decreased PSII activity (Fig. 4C), which correlated with a progressive loss of PsbA (Supplementary Fig. S4). The largest decrease of PSII activity occurred in LHCSR3-deficient *npq4* and the smallest decrease in WT-D66, the WT with the highest level of LHCSR3 (Fig. 4A). After 1–5 h recovery, *F*_v_/*F*_m_ values of cultures light treated in the presence of lincomycin decreased in both N_2_ and O_2_ purging to values of 0.33–0.36 and 0.10–0.28, respectively (Fig. 4C).

To investigate the repair process of PSII, *npq4*, WT-4A, and WT-D66 were treated with an even higher light intensity (500 µmol m^−2^ s^−1^) in photoautotrophic liquid medium for 16 h without gas purging (ambient O_2_), which induced a loss of PsbA in *npq4* (Supplementary Fig. S5). Immediately after light treatment, the quantum yield of PSII of *npq4* was <0.2, which increased along with increased PsbA levels during 1 h recovery. The *F*_v_/*F*_m_ of *npq4* reached WT values within 4 h when recovered in the presence of light, but not in the dark (Supplementary Fig. S5). In WT-D66, light had no effect on the recovery of *F*_v_/*F*_m_, showing that synthesis of D1 protein, reassembly of the PSII reaction centre, and photoactivation (i.e. light-dependent assembly of the Mn cluster) were not needed. Light influenced the recovery of *F*_m_ in WT-4A, showing some photoinhibition under these conditions, but less than occurred in *npq4* (Supplementary Fig. S5). Nonetheless, a rapid increase of *F*_m_ occurred in *npq4* when recovery was under low light, indicating an efficient PSII repair cycle.

### Light stress-associated production of reactive electrophile species and other aldehydes

**Fig. 5.**
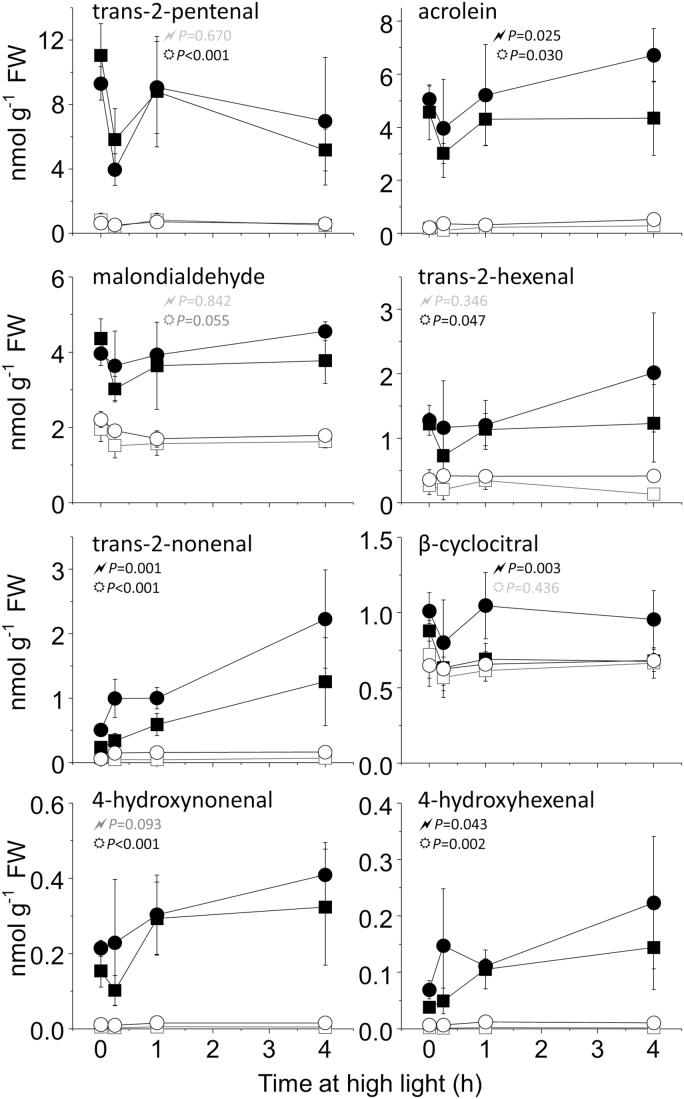
Light stress is associated with production of RES. WT-4A (squares) and *npq4* (circles) were grown photoautotrophically (filled symbols) or mixotrophically (open symbols) on 1.5% agar plates at 50 µmol photons m^−2^ s^−1^ before exposure to high light (250 µmol photons m^−2^ s^−1^). 2,4-DNPH-derivatized RES were measured by LC-MS/MS. The *P*-values from a two-way ANOVA of interaction between ‘genotype and media’ and ‘genotype and high light exposure time’ are represented by ‘
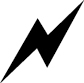
’ and ‘
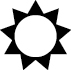
’, respectively, in black (*P*<0.05) or grey (*P*>0.05), *n*=4±SD.

**Fig. 6.**
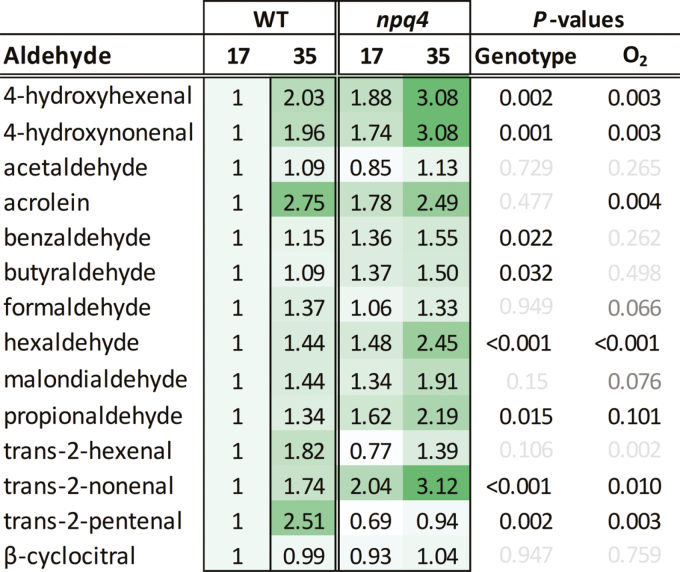
High oxygen tensions during culturing lead to higher RES and other aldehyde levels, and more in *npq4* than in the WT. Cells were grown photoautotrophically on 1.5% agar under photoautotrophic conditions in a 17% or 35% O_2_ atmosphere for 5 d under a 16/8 h light/dark cycle (250 µmol photons m^−2^ s^−1^). 2,4-DNPH-derivatized aldehydes, including several RES, were measured by LC-MS/MS, normalized to dry weight, and shown as fold change relative to the WT at 17% O_2_, as also indicated by shading. For each aldehyde, the *P*-values from a two-way ANOVA of genotype or percentage O_2_ are given in black (*P*<0.05) or grey (*P*>0.05), *n*=4±SD. (This figure is available in colour at *JXB* online.)

Photoautotrophic *C. reinhardtii* cells are under more light stress and produce more ^1^O_2_ compared with mixotrophic cells grown with an organic carbon source, such as acetate (Roach et al., 2013, 2017). Aldehydes and carbonyls, including RES, are produced under light stress as downstream products of lipid peroxidation (Mano, 2012). The concentrations of RES were elevated in photoautotrophic cells compared with mixotrophic cells, both before and during 4 h high light treatment (Fig. 5). Overall, *npq4* produced a higher level of RES than the WT, with an ANOVA revealing a significant interaction for genotype (*npq4* or WT-4A) and medium (photoautotrophic or mixotrophic) for acrolein, *trans*-2-nonenal, β-cyclocitral, and 4-hydroxyhexanal. Levels of aldehydes, including the aforementioned RES, were also measured in *npq4* or WT-4A cultivated in 17% or 35% O_2_, under the same conditions used in Fig. 3. For most aldehydes, cultivation in 35% O_2_ led to a significantly higher level than growth in 17% O_2_, and half of the determined aldehydes were significantly higher in *npq4* than in the WT (Fig. 6). Exogenously treating cells with acrolein for 4 h showed a concentration-dependent loss of maximum P700^+^ levels (Supplementary Fig. S6), revealing that it affects PSI. Furthermore, treating WT cells for 3 h with high light in the presence of the PSII inhibitor bromoxynil led to a slight, but significant (α=0.026, *t*-test), decline in maximum P700^+^ levels relative to dichlorophenyl dimethylurea (DCMU) treatment (Supplementary Fig. S6), whereby bromoxynil promotes more ^1^O_2_ production within PSII than does DCMU (Fufezan *et al.*, 2002). RES are also signalling molecules that contribute to ^1^O_2_ signalling. For example, acrolein and other RES induced tolerance to the ^1^O_2_-producing photosensitizer Rose Bengal (Fischer *et al.*, 2012; Roach *et al.*, 2018). Treating cultures with high light significantly enhanced tolerance of *npq4* to Rose Bengal more than WT-4A, while high light treatment in elevated O_2_ further induced Rose Bengal tolerance (Fig. S7).

### Acrolein-induced changes in photosynthesis-related transcription

**Fig. 7.**
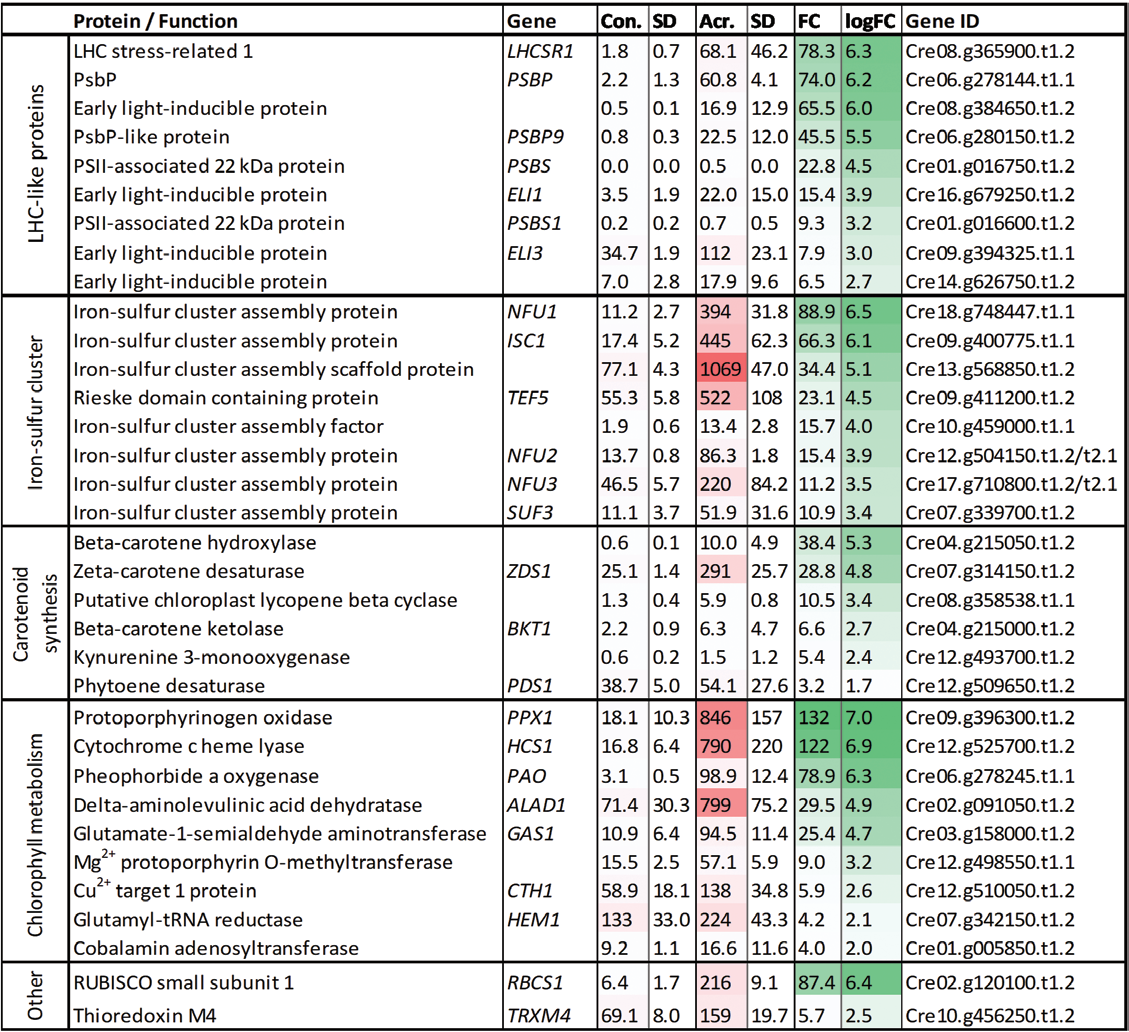
Transcription affected by the RES acrolein associated with NPQ, carotenoid synthesis, iron–sulfur clusters, and other selected chloroplast-associated pathways. Data were retrieved from RNA-seq of agar-grown cells treated with 600 ppm (atmospheric) volatile acrolein (Acr.) compared with non-treated cells (Con.) (Roach *et al.*, 2018). Intensity of shading indicates the relative reads per kilobase of transcript per million mapped reads (RPKM; dark shading=higher value) and fold changes (FC). All listed genes have significant differential expression (false-discovery-rate *P*<0.01) relative to control, *n*=3. (This figure is available in colour at *JXB* online.)

Previously, an RNA sequencing (RNA-seq) analysis of acrolein-treated *C. reinhardtii* focused upon the up-regulation of genes involved in redox defences, specifically thiol–disulfide exchanges (Roach *et al.*, 2018). Acrolein has been used as an example RES, but, most probably, the overall RES load is important, since various RES are able to activate the same ERE transcription factors (Fischer *et al.*, 2013). Further investigation of the RNA-seq data set of Roach *et al.* (2018) shows that up-regulated genes in response to a non-toxic acrolein dose include *LHCSR1* (78-fold) and *PSBS* (23-fold), as well as transcripts encoding several early light-inducible proteins (ELIPs) that are involved in photosystem assembly (Beck *et al.*, 2017), and proteins for carotenoid biosynthesis (Fig. 7). Furthermore, many iron–sulfur assembly protein-encoding genes were significantly up-regulated, such as *NFU1* (89-fold), *ISC1* (66-fold), and *TEF5* (23-fold).

## Discussion

The production of ROS is inevitable during photosynthesis, and dissipation of excess light energy via NPQ prevents further ROS formation. Photoinhibition of PSII, here defined as a decrease in maximal O_2_ production and PsbA levels, only occurred at a high O_2_ tension (Fig. 4), indicating that ROS production is an important contributor to this process. The higher sensitivity of *npq4* to photoinhibition in elevated O_2_ further supports previous observations that qE, and especially LHCSR3, prevents ROS production (Allorent *et al.*, 2013; Roach *et al.*, 2015a). Singlet oxygen has been shown to be the major ROS involved in photooxidative damage to plants (Triantaphylidès *et al.*, 2008), but evidently other ROS and RES also attack PSII (Chan *et al.*, 2012; Kale *et al.*, 2017; Roach *et al.*, 2017).

PSII is a particularly labile protein complex, which has a very efficient repair mechanism (Lidholm *et al.*, 1987; Aro *et al.*, 1993; Vass, 2012). Protein synthesis in the chloroplast, including the D1 reaction centre of PSII, has been shown to be inhibited by ROS, contributing to photoinhibition (Nishiyama *et al.*, 2001; Murata *et al.*, 2007). However, *npq4* maintained a high level of PSII repair (Supplementary Fig. S5), responding to a 5-fold increase in light intensity by increasing net O_2_ production (Supplementary Fig. S2). In the absence of repair (with lincomycin), PSII activity was completely lost within 2 h (Supplementary Fig. S2). Photoinhibition is often measured by a reduction in *F*_v_/*F*_m_ values, which can occur from a lower *F*_m_ or higher *F*_o_ value. In green algae, such as *C. reinhardtii*, a high level of state transitions (qT) can cause major changes in *F*_m_ that are not due to photoinhibition (Allorent *et al.*, 2013; Roach and Na, 2017). In addition, other types of sustained quenching exist, which cannot be attributed to either qT, photoinhibition of PSII (qI), or qE (Brooks *et al.*, 2013). High light treatment in the absence of O_2_ induced a sustained quenching of chlorophyll fluorescence, leading to lowered *F*_v_/*F*_m_ values (Fig. 4C). However, this did not correspond to lowered PSII activity (Fig. 4B), and is therefore not qI. A strong quenching of *F*_m_ in anaerobic conditions, which did not correlate with a loss of O_2_ evolution, was also observed in thylakoid membranes from higher plants (Kirilovsky *et al.*, 1994). In *A. thaliana*, a sustained non-qI quenching localized to the peripheral antenna (LHCII) of PSII, involving a plastid lipocalin (LCNP), was described as qH (Malnoë *et al*., 2018). Under cold and high light stress, mutants either deficient in or overexpressing LCNP produced more or fewer lipid peroxides than the WT, respectively, showing that qH is photoprotective (Malnoe *et al*., 2018). Here, light treatment in the absence of O_2_ led to ~40% lower *F*_v_/*F*_m_ values after 1.5 h recovery, despite the fact that the maximal PSII activity was not affected (Fig. 4B). Therefore, we propose that a similar qH quenching mechanism exists in *C. reinhardtii*, although probably via a different protein, since a BLAST search in *C. reinhardtii* failed to reveal any sequence similarity to LCNP. Since this O_2_-independent quenching occurred equally in both WTs and *npq4* (Fig. 4C), it is independent of LHCSR3. Sustained quenching in plants can also occur from retained zeaxanthin in LHC, termed qZ (Verhoeven *et al.*, 1996; Nilkens *et al.*, 2010), but in *C. reinhardtii* zeaxanthin is not a major contributor to chlorophyll fluorescence quenching (Quaas *et al.*, 2015).

Historically, atmospheric oxygen peaked ~300 mya at a level of 30–35% (Holland, 2006), but it is noteworthy that some algae living today in an atmosphere of 21% O_2_ have to tolerate much higher O_2_ tensions in highly oxygenated water columns. Water-borne photosynthetic organisms can raise O_2_ concentrations, which coincide with elevated H_2_O_2_ concentrations and the highest qE levels (Roach *et al.*, 2015b). *Chlamydomonas reinhardtii* increased NPQ-related protein levels in response to elevated oxygen tensions (Fig. 3A). In 35% O_2_, the *npq4* mutant compensated for the lack of LHCSR3 by elevating LHCSR1, which probably contributed to its increased qE capacity induced by high light at high O_2_ tensions. However, the qE capacity was always compromised in the *npq4* mutant by at least 2-fold relative to the WT (Figs 1, 2).

In contrast to PSII, PSI has a less efficient repair system (Sonoike, 2011). Under an elevated light intensity, *C. reinhardtii* responds by decreasing PSI levels, including the PSI antenna (Bonente *et al.*, 2012). The protection afforded by LHCSR3 to PSI at high oxygen tensions (Fig. 3) is intriguing since the protein interacts with LHCII, the major antenna of PSII (Tokutsu and Minagawa, 2013). An over-reduced plastoquinone pool, as could be expected in *npq4* under high light, activates phosphorylation of LHCII that can subsequently migrate to PSI during qT (Lemeille *et al.*, 2010). After a transition to state II, a high proportion of LHCII becomes an antenna of PSI (Drop *et al.*, 2014). LHCSR3 has been identified in the PSI antenna of *C. reinhardtii* (Allorent *et al.*, 2013; Bergner *et al.*, 2015), where it may potentially quench the excitation energy of LHCII, thereby decreasing the excitation pressure of PSI, as previously shown in the moss *P. patens* (Pinnola *et al.*, 2015) and *C. reinhardtii* (Girolomoni *et al.*, 2019). It should also be considered that LHCSR3 may also indirectly affect PSI activity in *C. reinhardtii* by, for example, affecting other photoprotective mechanisms, such as qT (Roach and Na, 2017), or preventing PSII-derived ^1^O_2_ formation and the downstream production of RES.

Previously, we have shown that *npq4* suffers from more photoinhibition than WT-4A when cultivated on photoautotrophic agar medium, and has higher levels of protein carbonylation (Roach *et al.*, 2017). This agrees with the higher RES levels detected in photoautotrophic *npq4* compared with WT-4A (Fig. 5). Acrolein treatment damaged PSI (Supplementary Fig. S6; Roach *et al.*, 2017), possibly targeting the iron–sulfur clusters of PSI, which are known to be damaged in PSI photoinhibition (Sonoike *et al.*, 1995; Tiwari *et al.*, 2016). The *npq4* mutant showed the tendency of higher RES production on photoautotrophic media, especially when cultivated in 35% O_2_ (Fig. 6), alongside a loss of photoinducible P700^+^ formation (Fig. 3D). Therefore, we suggest that RES may contribute to PSI photoinhibition.

**Fig. 8.**
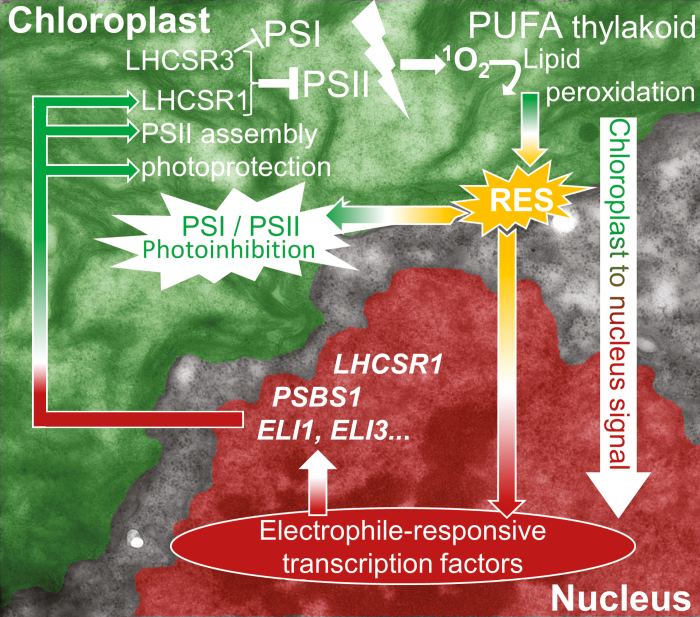
The involvement of reactive electrophile species (RES) in the Jekyll and Hyde high light stress responses of *C. reinhardtii*. Excess light increases the formation of singlet oxygen (^1^O_2_) from PSII, which can induce lipid peroxidation of the thylakoid membrane lipids in the chloroplast (green). Lipid peroxides decay to release RES (yellow) that attack photosystems, contributing to photoinhibition, but are also sensed by specific nuclear transcription factors, such as SOR1, to affect transcription in the nucleus (red), whereby RES act as chloroplast-to-nucleus ‘retrograde’ signals. Up-regulated transcripts (white italics) are involved in helping mitigate excess light energy (*LHCSR1*, *PSBS1*), or contribute to photosystem assembly (*ELI1*, *ELI3*). The pathway, modified with permission from Roach *et al.* (2018) is superimposed over a false-coloured electron micrograph of an algal cell. The non-coloured region is the cytosol.

It is increasingly accepted that RES also contribute to stress signalling and can be involved in ROS signal propagation (Roach *et al.*, 2018; Mano *et al.*, 2019). Treatment of WT cells with acrolein increased the expression of genes involved in iron–sulfur cluster assembly (Fig. 7). Other up-regulated genes included *LHCSR1* (78-fold) and *PSBS1* (23-fold), which both function in qE (Allorent *et al.*, 2016; Dinc *et al.*, 2016; Tilbrook *et al.*, 2016), helping mitigate light stress and prevent further RES production. Higher RES levels would explain why *npq4* and the WT contained more LHCSR1 and PsbS (in the WT) when cultivated in 35% O_2_ (Figs 3A, 6). As summarized by Fig. 8, on the one hand RES cause damage in the chloroplast, attacking proteins and contributing to photoinhibition, while on the other hand function in ^1^O_2_-mediated chloroplast to nucleus signalling during high light acclimation.

### Conclusions

We showed that O_2_ availability influences how important qE is in protecting the photosynthetic apparatus from photoinhibition, and that in *C. reinhardtii* the O_2_ tension is a regulator of qE capacity. This may partially be due to the elevated levels of RES, which up-regulated expression of *LHCSR1* and *PSBS*. Since *npq4* was suffering a heavier RES load at elevated O_2_ concentrations, LHCSR3 protects against oxygen-mediated stress. This may include PSI photoinhibition, although the relative contribution of RES to this remains to be elucidated, as LHCSR3 also quenches excitation energy in the PSI antenna. Even though the PSII repair cycle was sufficient to prevent a net loss of PSII levels in *npq4*, elevated PSII repair rates would have energetic costs that may also affect growth rates in elevated O_2_ tensions. The lack of indifference in phenotype between *npq4* and the WT light treated with low O_2_ availability shows that a multilevel qE mechanism was only necessary when O_2_ was highly abundant. Therefore, an O_2_-rich atmosphere can be considered a potential driver in the evolution of qE mechanisms.

## Supplementary data

Supplementary data are available at *JXB* online.

Fig. S1. Influence of 35% or 17% O_2_ during growth on levels of PsaA and PsbA protein levels in WT-4A and *npq4*.

Fig. S2. Effect of high light in the presence and absence of lincomycin on maximum PSII activity in WT-4A and *npq4*.

Fig. S3. Effect of high light on NPQ and LHCSR1 levels in WT-4A and *npq4*.

Fig. S4. Changes in PsbA protein levels in WT-4A and *npq4* during high light treatment in the presence of lincomycin with N_2_ or O_2_ gas purging.

Fig. S5. The effect of light during post-high light recovery on relative and PSII quantum yields and PsaA protein levels in WT-D66 and *npq4*.

Fig. S6. Effect of acrolein, bromoxynil, and DCMU on P700-dependent absorption changes, indicating maximum P700^+^ levels.

Fig. S7. High light-treated *npq4* has higher tolerance to ^1^O_2_ than the WT, and high light treatment in a high O_2_ atmosphere induces greater tolerance than in ambient O_2_.

eraa022_suppl_supplementary_figures_S1_S7
