# Supplementary material for: The non-photochemical quenching protein LHCSR3 prevents oxygen-dependent photoinhibition in Chlamydomonas reinhardtii
Source: J Exp Bot. 2020 Jan 16;71(9):2650–60. doi: 10.1093/jxb/eraa022 (PMC7210768; doi:10.1093/jxb/eraa022)
Supplement: eraa022_suppl_supplementary_figures_S1_S7 [file eraa022_suppl_supplementary_figures_s1_s7.pdf]

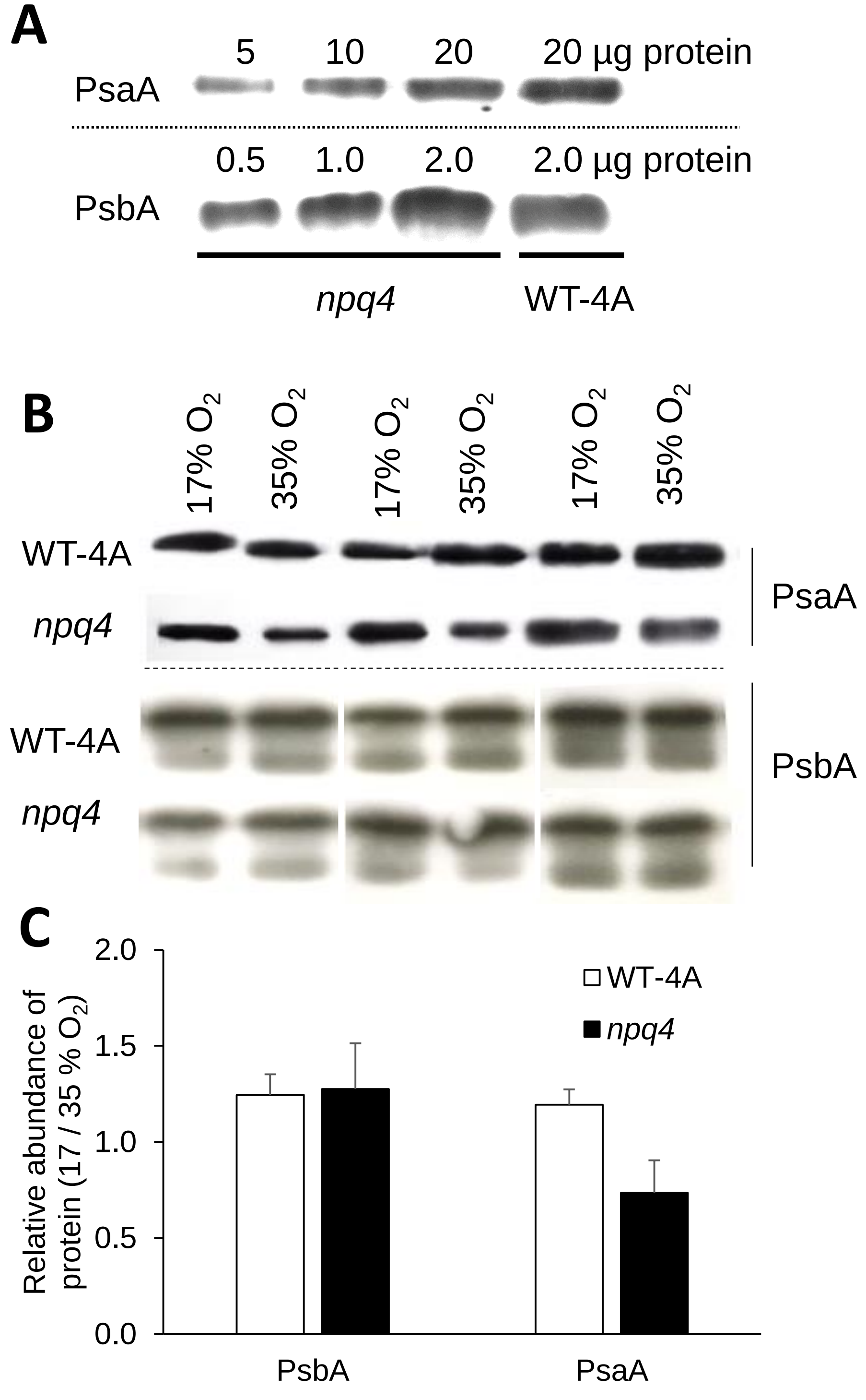

Supplemental Figure 1. Influence of O_2 on levels of PSI and PSII reaction centres, PsaA and PsbA, respectively, as indicated by western blotting of *C. reinhardtii* wild-type (WT) 4A and *npq4* grown under 17% and 35% O_2. (A) Various protein amounts were loaded for checking suitable protein amounts for western blotting of PsbA and PsaA in cells grown under 35% O_2. (B) Protein extracts from cells grown under 17% and 35% O_2 were from three independent experiments conducted on three separate occasions, but loaded and blotted together on the same 15-well gel. Each lane contained 10µg protein for PsaA and 1µg protein PsbA. (C) Densitometric quantification of band intensity of data shown in (B) for WT-4A (white) and *npq4* (black).

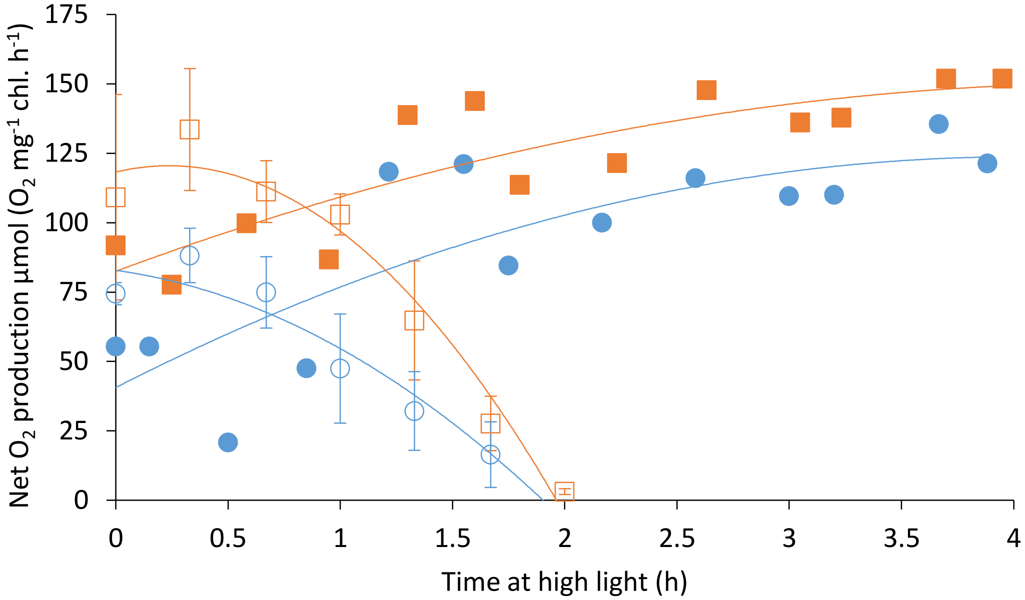

Supplemental Figure 2. Effect of high light in the presence and absence of lincomycin on PSII activity, as measured via net O_2 production under saturating light in the presence of 1 mM $NaHCO_3$. Closed symbols without lincomycin from two independent experiments measured at different time points. Open symbols with 2.5 mM lincomycin, n=3±SD. Orange and blue symbols represent wild type (WT)-4A and *npq4*, respectively.

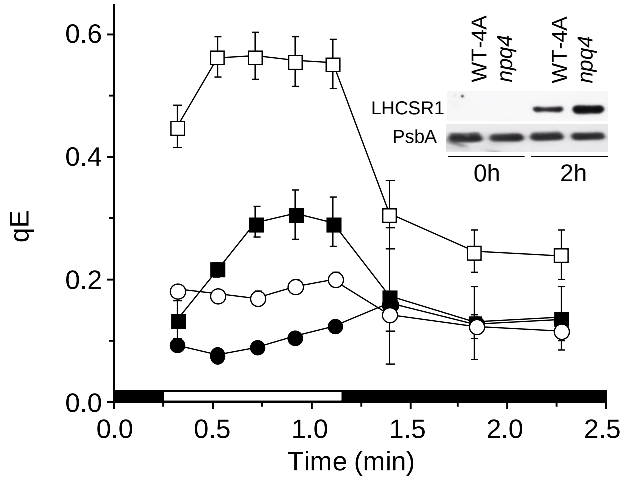

Supplemental Figure 3. Effect of high light on NPQ and LHCSR1 levels in WT-4A and *npq4*. NPQ at 1000 µmol quanta m^{-2} s^{-1} as indicated by the white bar on the x-axis. Wild-type 4A (WT; squares) and *npq4* (circles) were measured before (filled symbols) or after a high light-treated for 2 h (+1.5 h recovery) n=3±SD. The inset shows a western blot of the NPQ protein LHCSR1 in the same cells used for NPQ measurements, with PsbA (PSII reaction centre) shown as loading control.

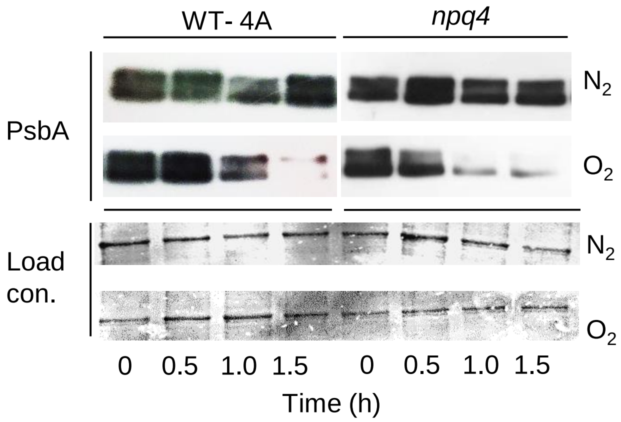

Supplemental Figure 4. **Changes in PsbA protein levels in *npq4* and WT during high light treatment in the presence of lincomycin with N_2 or O_2 gas purging**. Wild-type (WT)-4A and *npq4* were high light-treated for 1.5 h in the presence of 2.5 mM lincomycin and 5mM $NaHCO_3$ while purging with N_2 or O_2 gas. 1µg of protein was loaded per lane. The Coomassie-stained band of the large RuBisCo subunit (*c*. 50kD) is provided for loading controls. Shown are typical blots from three independent experiments of proteins extracted from the same cells used for data shown in Figure 4.

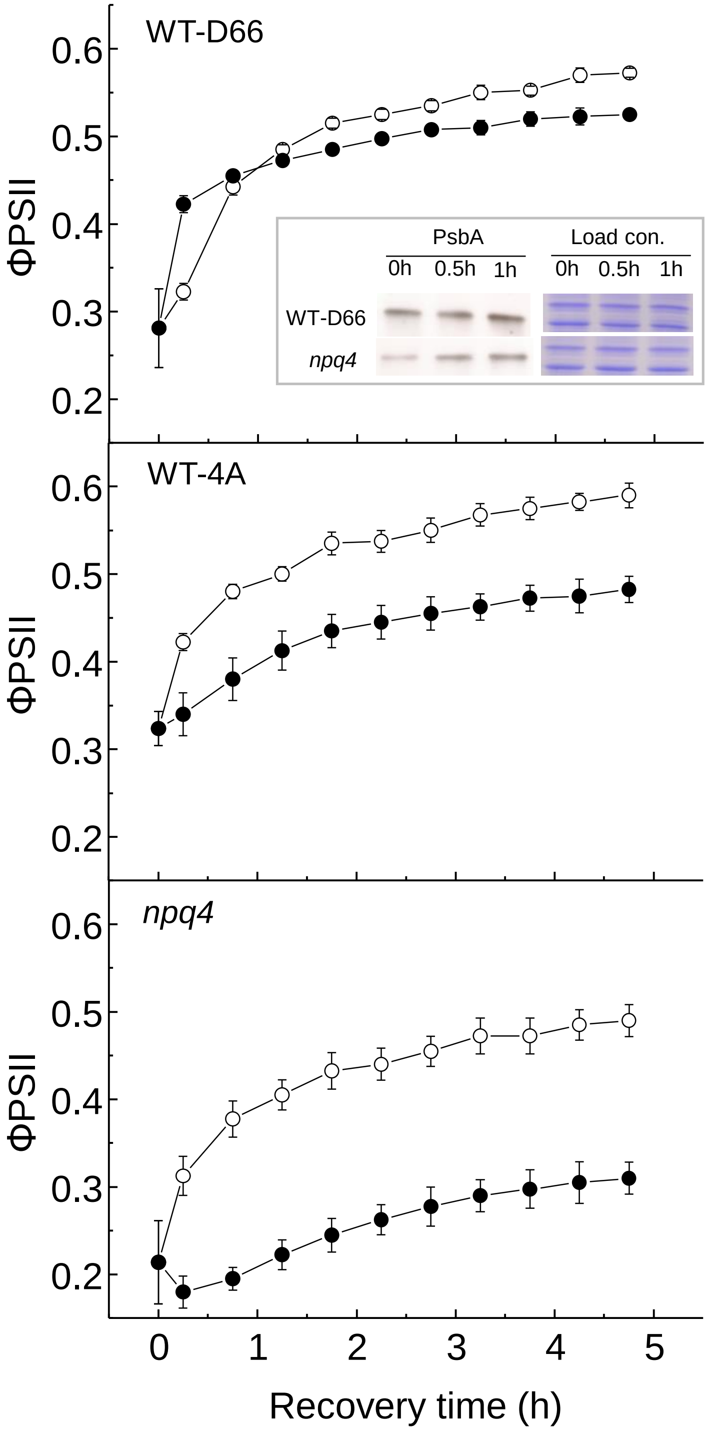

Supplemental Figure 5. **The effect of low light during recovery of high light-treated cultures on quantum yields of PSII (ΦPSII) and levels of PsbA, the PSII reaction centre**. WT-D66, WT-4A and *npq4* were treated for 16 h under high light (500 µmol m^{-2} s^{-1}) in photoautotrophic liquid media and then recovered in the dark (black) or low light (25 µmol quanta m^{-2} s^{-1}; white), n = 3 ± SD. The difference in ΦPSII between dark and low light recovered cells is indicative of photoinhibition. The inset in (A) shows PsbA levels of WT-D66 and *npq4* during recovery under low light. Coomassie-stained gel of the major LHC proteins (*c*. 20-28kD) is shown for loading control.

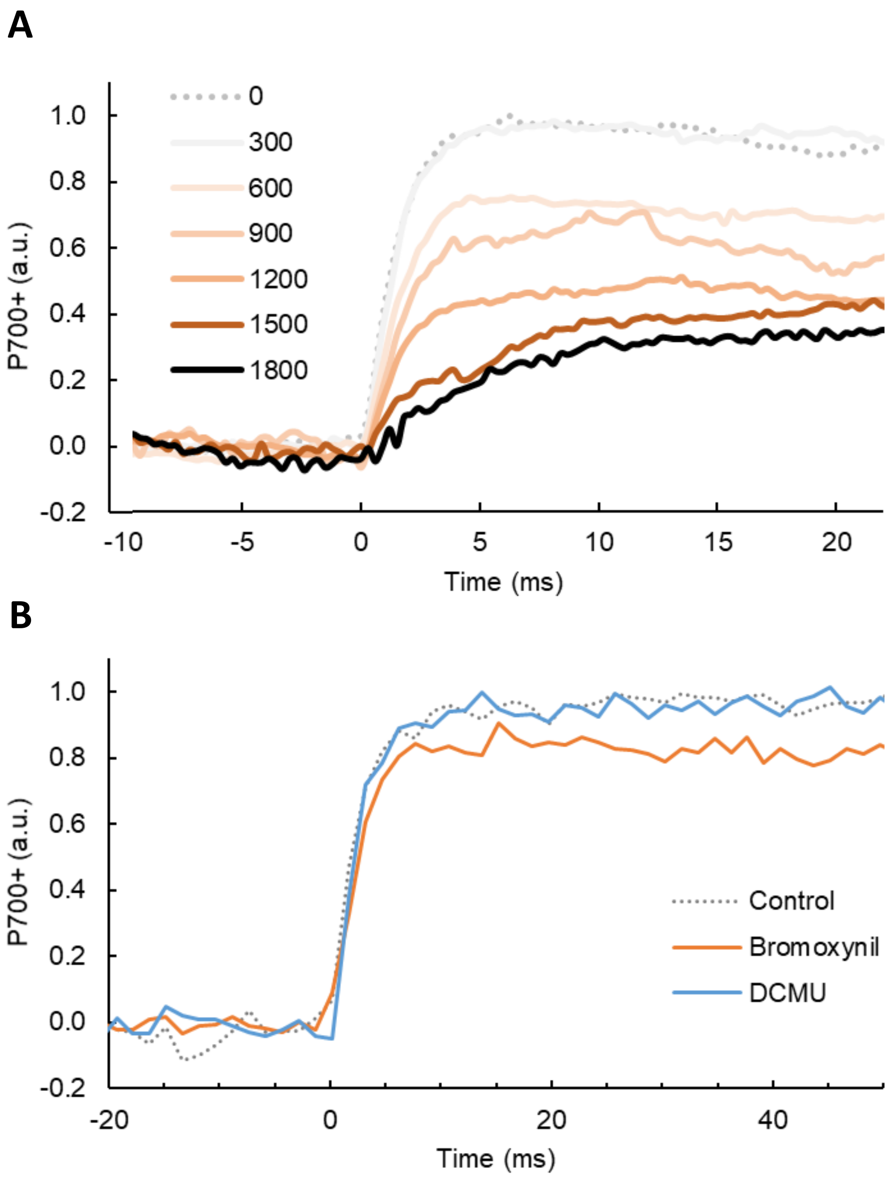

Supplemental Figure 6. Effect of acrolein, bromoxynil and DCMU on P700-dependent absorption changes, indicating maximum P700+ levels. (A) Cells were cultivated on TAP agar under low light before treatment with 0-1800 ppm (atmospheric) concentrations of volatile acrolein for 4 h, as indicated by the key. Kinetics are averages of three biological replicates each measured three times around a saturating pulse starting a 0 ms. Cells were cultivated on TAP agar under low light before treatment with 0-1800 ppm (atmospheric) concentrations of volatile acrolein for 4 h, as indicated by the key. (B) Photoautotrophic cells were cultivated in liquid media (control; grey dotted line) and high light-treated at 300µE for 3 h in the presence of 50µM bromoxynil (orange line) or 10µM DCMU (blue line). All measurements were made in the presence of 10µM DCMU.

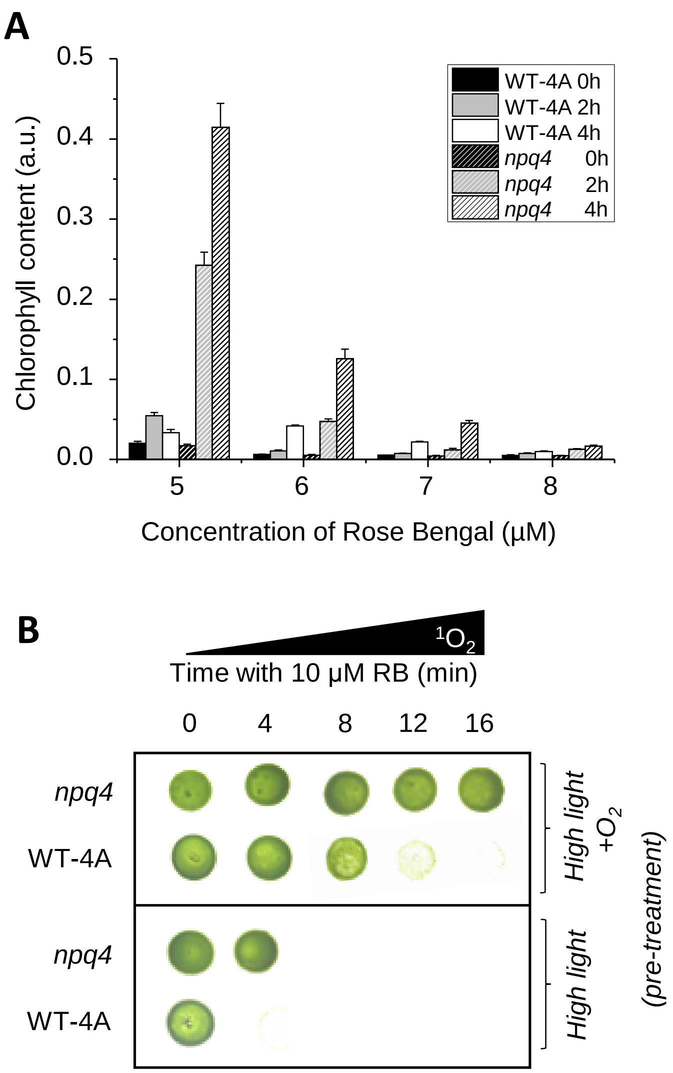

Supplemental Figure 7. **High light-treated *npq4* has higher tolerance to $^1O_2$ than WT, and high light treatment in a high O_2 atmosphere induces greater tolerance than in ambient O_2.** (A) WT-4A and *npq4* cells were treated with high light (300 µmol quanta m^{-2} s^{-1}) for 0, 2 or 4 h and tolerance to $^1O_2$ was tested by adding various concentrations of Rose Bengal (RB) for 24 h at 50 µmol quanta m^{-2} s^{-1}. Cell survival was monitored by chlorophyll content of cultures relative to non-treated cells, n=3±SD. (B) Photoautotrophic WT-4A and *npq4* cells on agar were pre-treated with high light for 4 h, either at ambient O_2 (high light) or 80 % O_2 (high light +O_2). Subsequently, cells were re-suspended in liquid medium at 10 µg mL^{-1} total chlorophyll with 10 µM RB and treated with high light for up to 16 min. Every 4 min, 10µL of RB-treated culture was spotted onto TAP agar and cultivated for 5 d under low light before imaging.
